# Supplementary material for: A genome-wide CRISPR screen identifies host factors that regulate SARS-CoV-2 entry
Source: Nat Commun. 2021 Feb 11;12:961. doi: 10.1038/s41467-021-21213-4 (PMC7878750; doi:10.1038/s41467-021-21213-4)
Supplement: Supplementary file 1 — Supplementary Information [file 41467_2021_21213_MOESM1_ESM.pdf]

A genome-wide CRISPR screen identifies host factors that regulate SARS-CoV-2 entry

Yunkai Zhu[1§], Fei Feng[1§], Gaowei Hu[1§], Yuyan Wang[1§], Yin Yu[1], Yuanfei Zhu[1], Wei Xu[1], Xia Cai[1], Zhiping Sun[1], Wendong Han[1], Rong Ye[1], Di Qu[1], Qiang Ding[2], Xinxin Huang[3], Hongjun Chen[4], Wei Xu[5], Youhua Xie[1], Qiliang Cai[1*], Zhenghong Yuan[1*], and Rong Zhang[1*]

Supplementary Figure and Figure legends 1-7

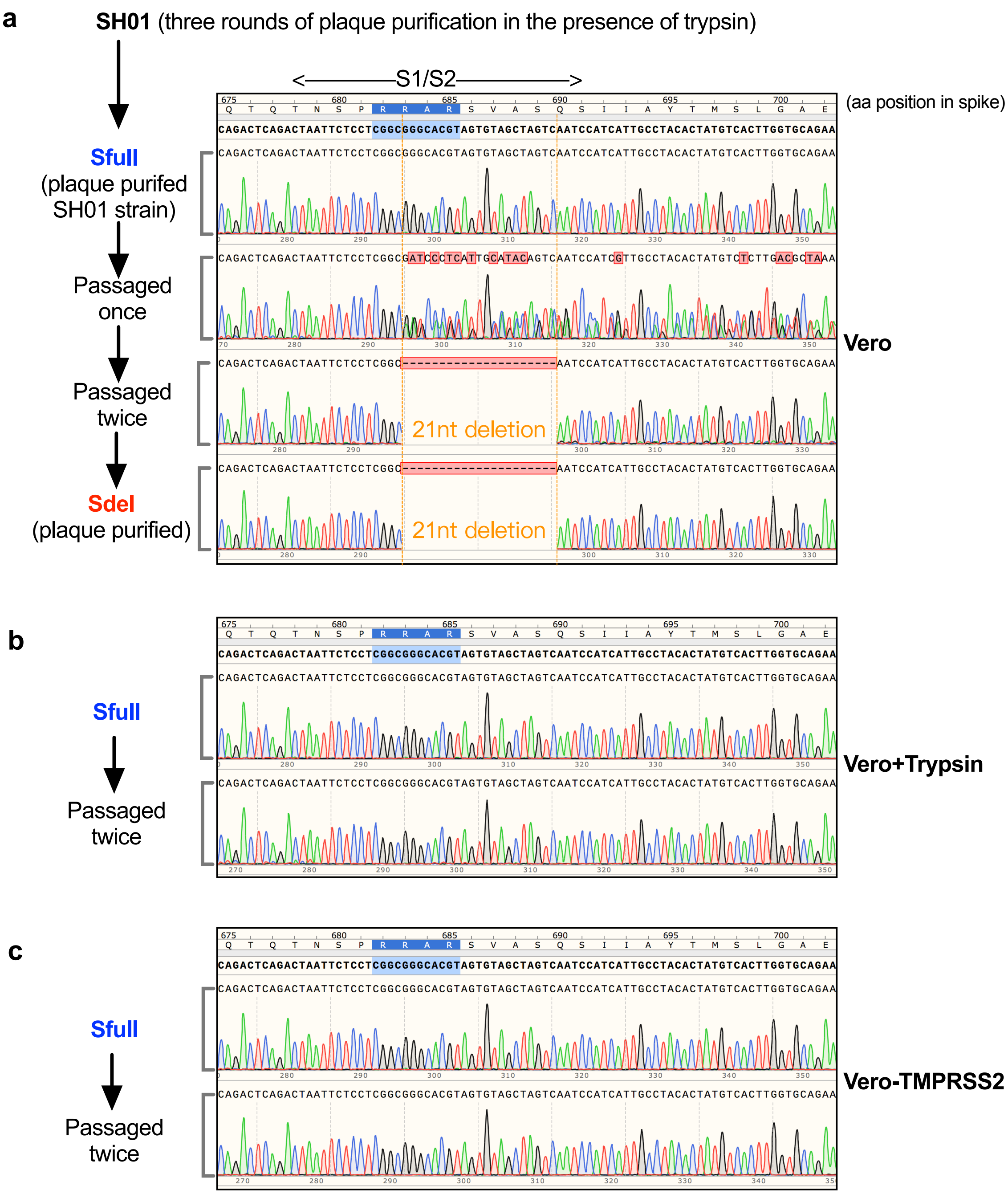

d

	nsp3 (nt 346)	nsp3 (nt 3307)	nsp8 (nt 382)	nsp14 (nt 1079)	Spike (nt 2048-2068)
**Wuhan-Hu-1**	G (Gly)	C (Pro)	C (Leu)	C (Ala)	-
**SH01**	G (Gly)	T (Ser)	T (Ser)	C (Ala)	-
**Sfull**	T (Cys)	T (Ser)	T (Ser)	T (Val)	-
**Sdel**	T (Cys)	T (Ser)	T (Ser)	T (Val)	21nt deletion (RARSVAS)

Supplementary Figure 1. The acquisition of Sfull and Sdel clones of SARS-CoV-2. **a.** SARS-CoV-2 SH01 strain isolated from a patient sample was purified three times by plaque assay on Vero-E6 (thereafter as Vero) cells in the presence of trypsin, resulting the clone of Sfull virus. The Sdel clone was obtained by passaging the Sfull virus twice and plaque-purified once on Vero cells without trypsin. The trace results of Sanger sequencing were generated by SnapGene Viewer, and the 21 nucleotide (nt) deletion was indicated. **b.** The Sfull strain was passaged twice on Vero cells in the presence of trypsin. **c.** The Sfull strain was passaged twice on Vero cells expressing the TMPRSS2 in the absence of trypsin. d. The sequence alignment of SARS-CoV-2 strains. The full-length genome sequences obtained by RT-PCR and Sanger sequencing were aligned and compared to the stain Wuhan-Hu-1. Wuhan-Hu-1, accession No. MN908947; SH01, accession No. MT121215.

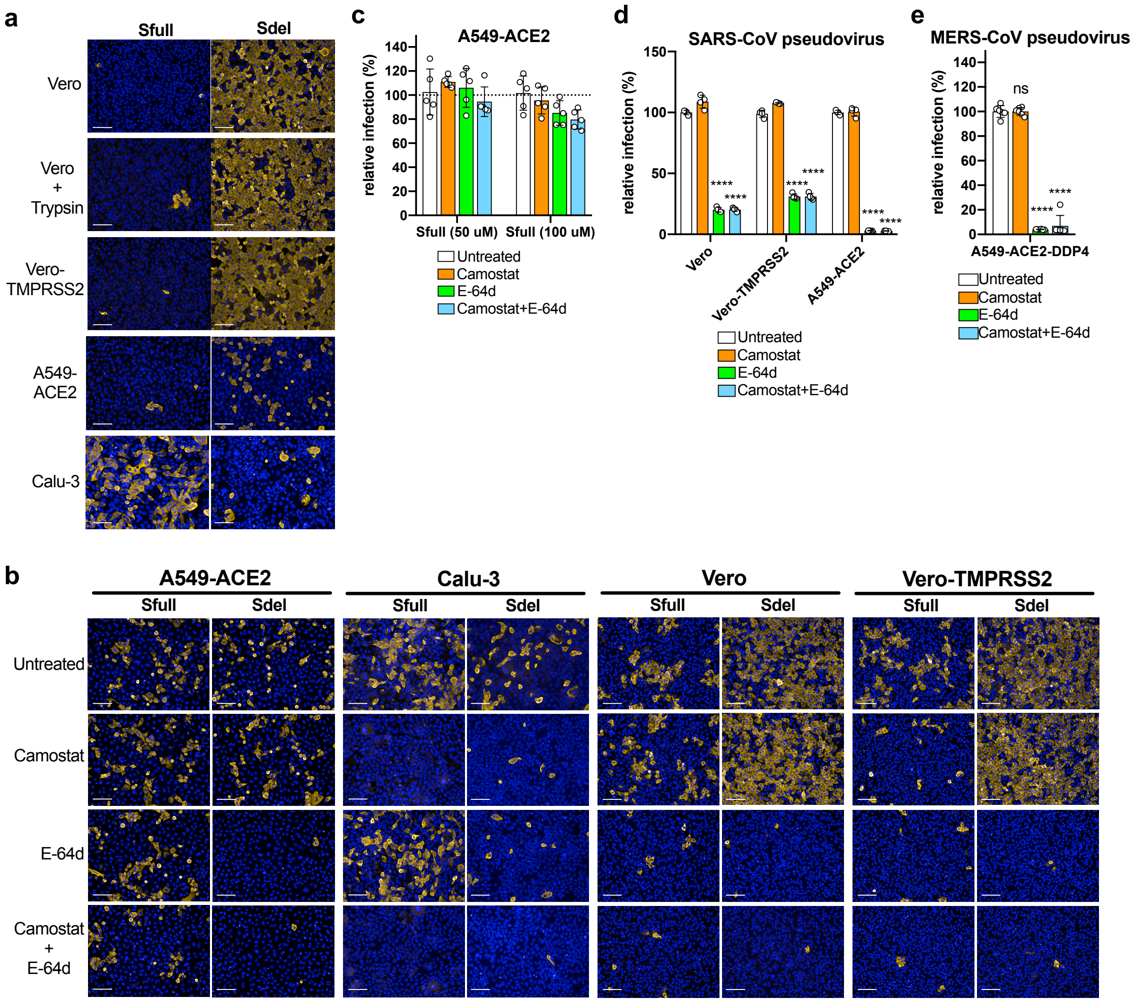

a
Sfull
Sdel
Vero
Vero + Trypsin
Vero-TMPRSS2
A549-ACE2
Calu-3
c
A549-ACE2
relative infection (%)
Sfull (50 uM)
Sfull (100 uM)
Untreated
Camostat
E-64d
Camostat+E-64d
d
SARS-CoV pseudovirus
relative infection (%)
Vero
Vero-TMPRSS2
A549-ACE2
****
Untreated
Camostat
E-64d
Camostat+E-64d
e
MERS-CoV pseudovirus
relative infection (%)
ns
****
A549-ACE2-DDP4
Untreated
Camostat
E-64d
Camostat+E-64d
b
A549-ACE2
Calu-3
Vero
Vero-TMPRSS2
Sfull
Sdel
Untreated
Camostat
E-64d
Camostat + E-64d

Supplementary Figure 2. The replication and entry property of Sfull and Sdel clones of SARS-CoV-2, SARS-CoV and MERS-CoV pseudoviruses. a. Immunofluoresence staining of the nucleocapsid (N) protein of Sfull or Sdel virus on wild type Vero, Vero with trypsin treatment, Vero expressing the TMPRSS2, A549-ACE2, and Calu-3 cells. Virus-infected cells were fixed, permeablized, and stained with the house-made mouse anti-nucleocapsid serum. After washing, cells were incubated with goat anti-mouse antibody conjugated with Alexa Fluor 555 (Thermo # A-21424, 2 μg/ml), followed by staining with 4',6-diamidino-2-phenylindole (DAPI). Images were collected using an Operetta High Content Imaging System (PerkinElmer), and processed using the ImageJ software. One representative image was shown from 2 independent experiments. Scale bar, 100μm. **b.** The effect of compounds Comostat and E-64d on the infection by Sfull or Sdel virus in different cell types. Cells were pretreated with 25 μM compounds Camostat or / and E-64d and infected with Sfull or Sdel virus in the presence of compounds for 24 h. Immunofluoresence assay was conducted as described above. One representative image was shown from 2 independent experiments. Scale bar, 100μm. **c.** Sfull infection on A549-ACE2 cells were resistant to the treatment of 50 μM or 100 μM of Comostat or / and E-64d. Cells were pretreated with the indicated compounds and infected with the Sfull virus in the presence of compounds, followed by Immunofluoresence staining as described above. **d-e.** The effect of compounds Comostat and E-64d on the infection by SARS-CoV or MERS-CoV pseudovirus in different cell types. Cells were pretreated with 25 μM compounds Camostat or / and E-64d and infected with Sfull or Sdel pseudovirus in the presence of compounds for 48 h. One-way ANOVA with Dunnett's test (c-e); n=5 (c), 4 (d), or 6 (e); 2 experiments; mean ± s.d.; ****$P < 0.0001$. Immunofluoresence assay was conducted as described above. Data were normalized to the controls of individual experiments.

a

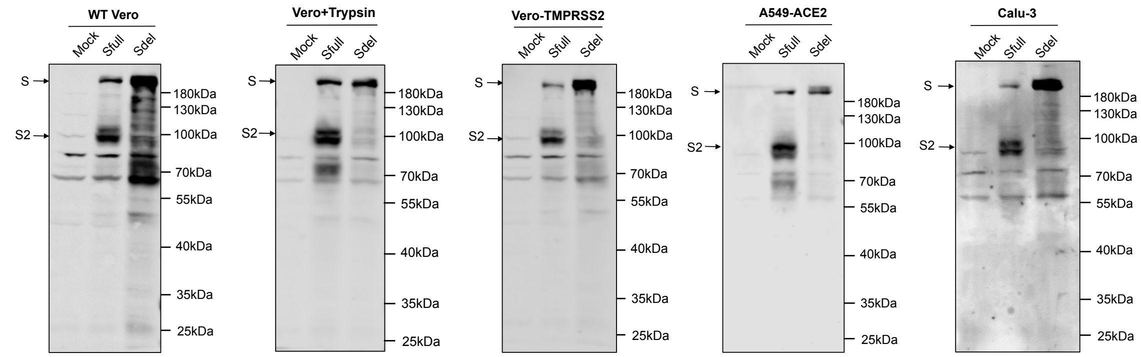
WT Vero
Mock
Sfull
Sdel
S
S2
180kDa
130kDa
100kDa
70kDa
55kDa
40kDa
35kDa
25kDa
Vero+Trypsin
Vero-TMPRSS2
A549-ACE2
Calu-3

b

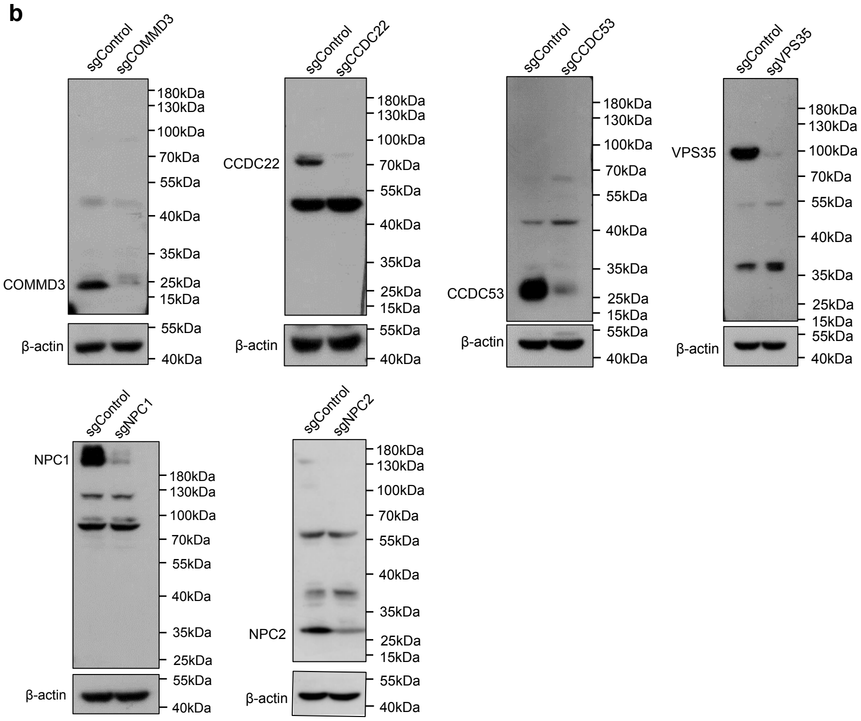
sgControl
sgCOMMD3
COMMD3
β-actin
sgCCDC22
CCDC22
sgCCDC53
CCDC53
sgVPS35
VPS35
sgNPC1
NPC1
sgNPC2
NPC2
180kDa
130kDa
100kDa
70kDa
55kDa
40kDa
35kDa
25kDa
15kDa

Supplementary Figure 3. The cleavage of spike protein in different cell types and editing efficiency of A549-ACE2 cells by CRISPR sgRNA of genes selected. a. Western blotting of cell lysates of different cell types or conditions inoculated with Sfull or Sdel virus. The cell lysates were probed with rabbit anti-SARS-CoV-2 spike S2 anbitody (Sino Biological #40590-T62), followed by incubating with horseradish peroxidase (HRP)-conjugated goat anti-rabbit polyclonal antibody and developed using SuperSignal West Pico chemiluminescent substrate. The bands corresponding to the full-length spike (S) and cleaved S2 subunit are indicated by arrows**. b.** Editing efficiency of A549-ACE2 cells by CRISPR sgRNA of genes selected. Genes in A549-ACE2 cells were edited by the indicated sgRNAs and the mixed population of cells was subjected to western blotting. Cell lysates were probed with rabbit anti-COMMD3 (proteintech #26240-1-AP), CCDC22 (proteintech #16636-1-AP), CCDC53 (proteintech #24445-1-AP), VPS35 (proteintech #10236-1-AP), NPC1 (proteintech #13926-1-AP), or NPC2 (proteintech #19888-1-AP) polyclonal antibody, followed by incubating with horseradish peroxidase (HRP)-conjugated goat anti-rabbit polyclonal antibody and developed using SuperSignal West Pico or Femto chemiluminescent substrate. One representative blot was shown from 2 independent experiments.

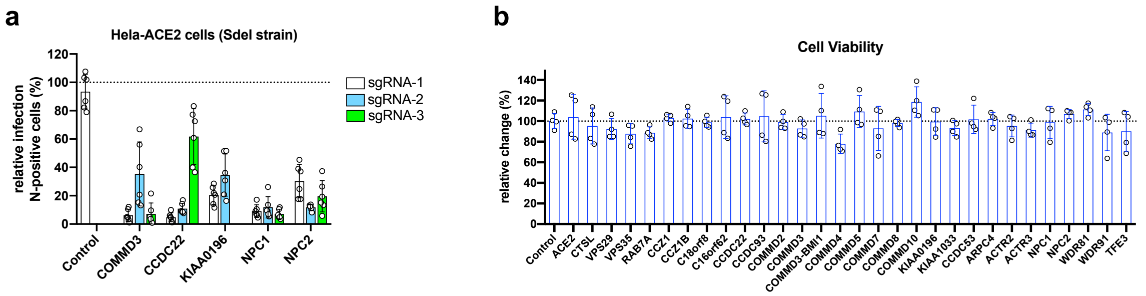

Supplementary Figure 4. Virus infection and cell viability in HeLa-ACE2 or A549-ACE2 cells. a. HeLa cells expressing the human ACE2 were edited by three different sgRNAs of selected genes. Cells were infected with Sdel virus and subjected to immunofluorescence assay and high-content imaging as described in Methods. Data were pooled from two independent experiments performed in triplicate, and are normalized to the controls of individual experiments. **b.** Viability of A549-ACE2 cells edited with individual CRISPR sgRNA of genes selected. An equal number of cells were plated and viability was assessed over a 48 h period using the Cell-Titer Glo assay. One-way ANOVA with Dunnett's test (a-b); n=6 (a) or 4 (b); 2 experiments; mean ± s.d..The results were normalized to control cells and are pooled from two independent experiments.

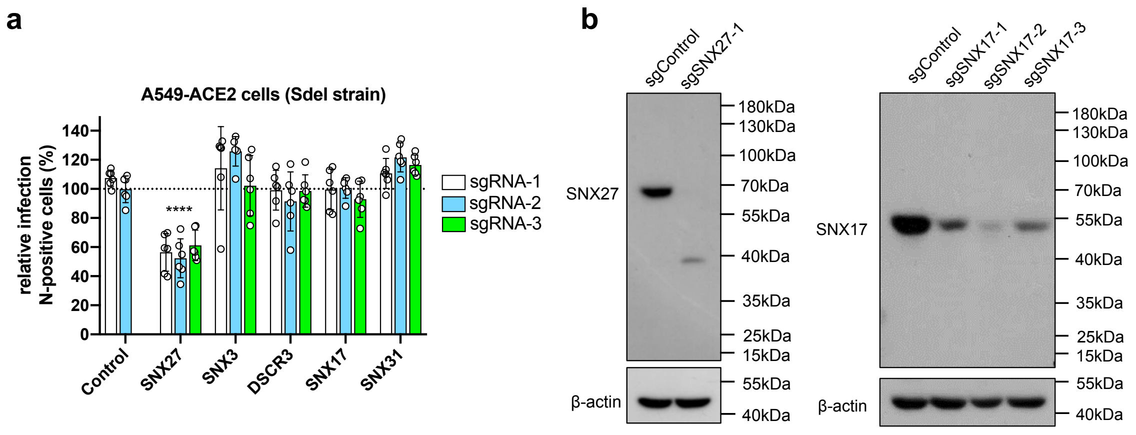

Supplementary Figure 5. Validation of genes related to cargo retrieval and recycling in A549-ACE2 cells. a. A549 cells expressing the human ACE2 were edited by three different sgRNAs of selected genes. Cells were infected with Sdel virus and subjected to immunofluorescence assay and high-content imaging as described in Methods. Data were pooled from two independent experiments performed in triplicate, and are normalized to the controls of individual experiments. One-way ANOVA with Dunnett's test; mean ± s.d.; ****P < 0.0001. **b.** Western blotting to confirm the editing efficiency in A549-ACE2 cells by the indicated sgRNAs. Cell lysates were probed with mouse anti-SNX27 (Abcam #ab77799) or rabbit anti-SNX17 (proteintech, #10275-1-AP) polyclonal antibody, followed by incubating with horseradish peroxidase (HRP)-conjugated goat anti-mouse or rabbit polyclonal antibody, and developed using SuperSignal West Pico or Femto chemiluminescent substrate.

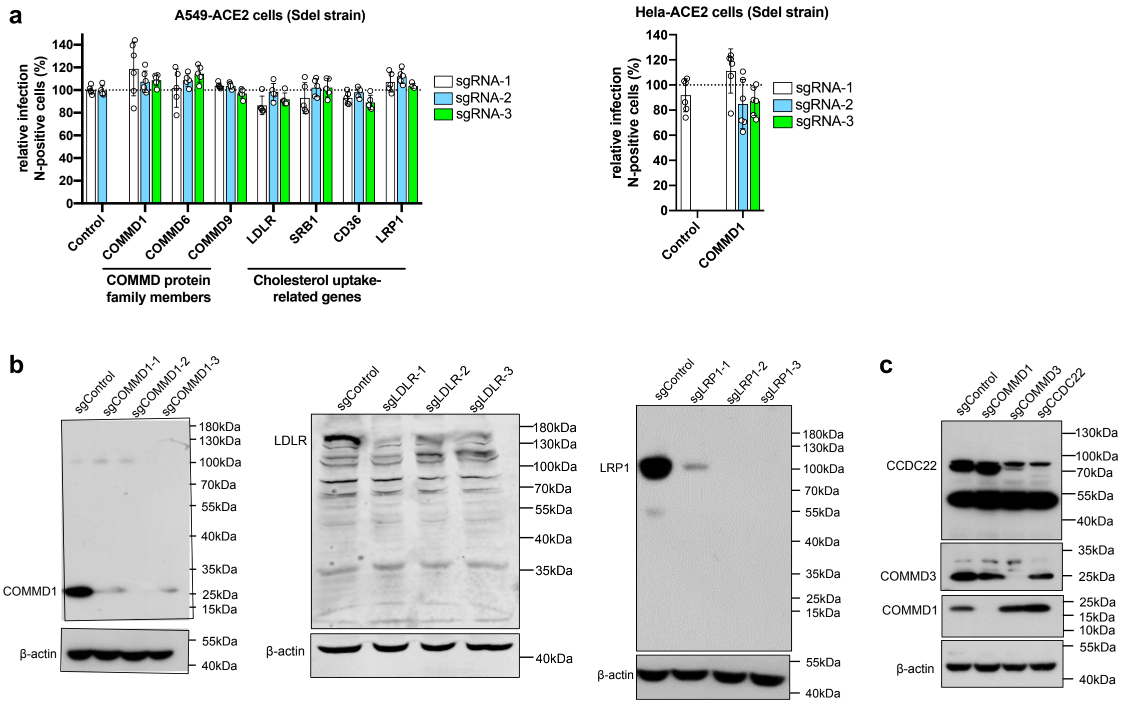

Supplementary Figure 6. Validation of COMMD protein family members and cholesterol uptake related genes in A549-ACE2 or HeLa-ACE2 cells. a. *COMMD1*, *6*, and *9*, and genes related to cholesterol uptake are not required for SdeI virus infection. A549 or HeLa cells expressing the human ACE2 were edited by three different sgRNAs of the indicated genes. Cells were infected with SdeI virus and subjected to immunofluorescence assay and high-content imaging as described in Methods (two experiments; one-way ANOVA with Dunnett's test; n=6 or 7; mean ± s.d.). Data were normalized to the controls of individual experiments. **b.** Western blotting to verify the editing efficiency of COMMD1, LDLR, and LRP1 genes in A549-ACE2 cells by the indicated sgRNAs. **c.** Western blotting to verify the expression of COMMD1, COMMD3, or CCDC22 affected by gene-editing. Cell lysated were probed with rabbit anti-COMMD1 (proteintech #11938-1-AP), LDLR (proteintech, #10785-1-AP), LRP1 (Abcam #ab92544), rabbit anti-COMMD3 (proteintech #26240-1-AP), or rabbit anti-CCDC22 (proteintech #16636-1-AP) polyclonal antibody, followed by incubating with horseradish

peroxidase (HRP)-conjugated goat anti-rabbit polyclonal antibody and developed using SuperSignal West Pico or Femto chemiluminescent substrate.

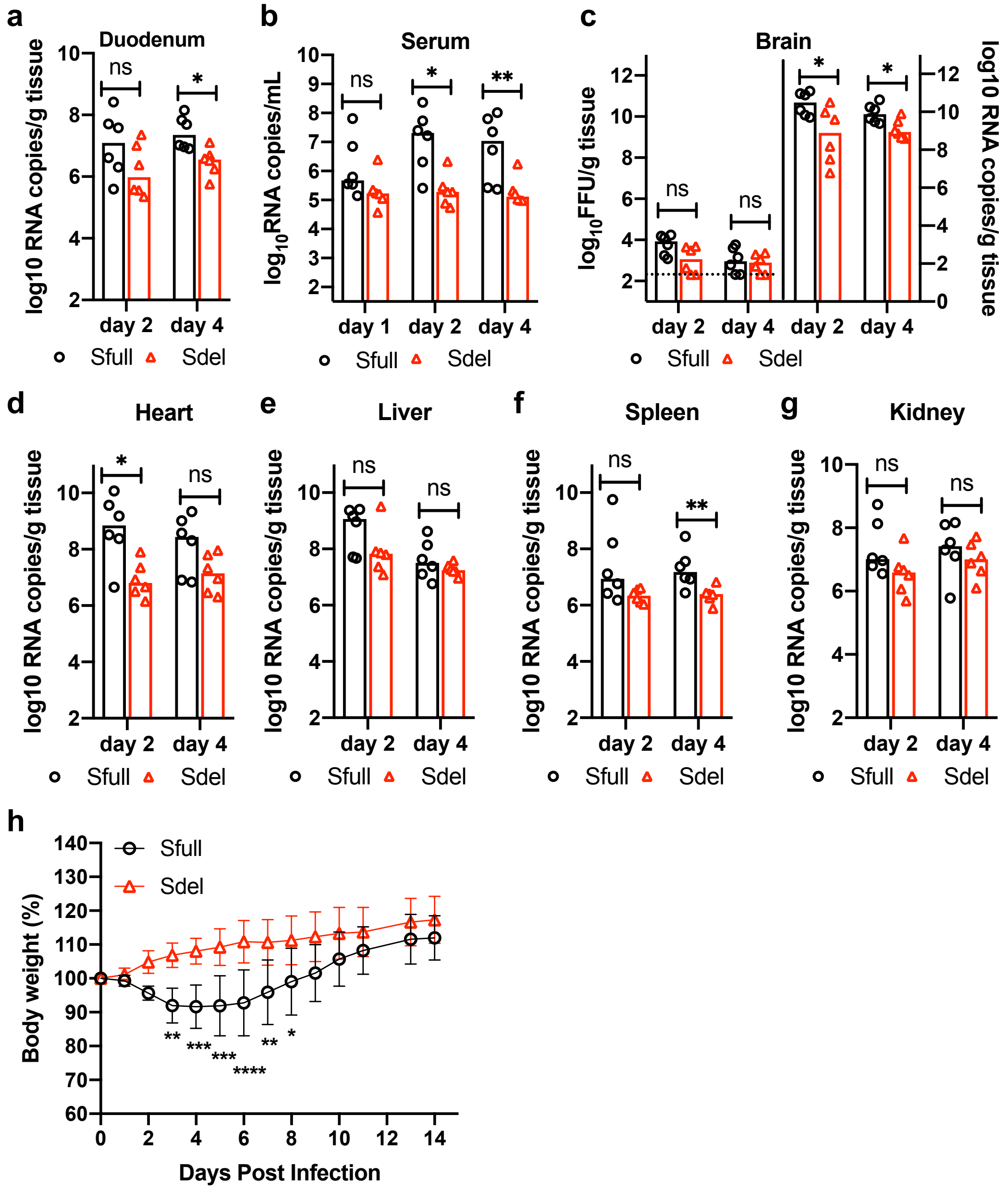

a
Duodenum
log10 RNA copies/g tissue
ns
*
day 2
day 4
Sfull
Sdel
b
Serum
log_{10}RNA copies/mL
ns
*
**
day 1
day 2
day 4
Sfull
Sdel
c
Brain
log_{10}FFU/g tissue
log10 RNA copies/g tissue
ns
ns
*
*
day 2
day 4
day 2
day 4
Sfull
Sdel
d
Heart
log10 RNA copies/g tissue
*
ns
day 2
day 4
Sfull
Sdel
e
Liver
log10 RNA copies/g tissue
ns
ns
day 2
day 4
Sfull
Sdel
f
Spleen
log10 RNA copies/g tissue
ns
**
day 2
day 4
Sfull
Sdel
g
Kidney
log10 RNA copies/g tissue
ns
ns
day 2
day 4
Sfull
Sdel
h
Sfull
Sdel
Body weight (%)
**
***
***
****
**
*
Days Post Infection

Supplementary Figure 7. Viral load in different tissues, body weight change and lung histology. a-g. 6-8 week-old hamsters were infected intranasally and serum (day 1, 2, 4) and tissues from intestine, brain, heart, liver, spleen, and kidney (day 2, 4) were harvested (n=6 per day). Viral RNAs were extracted for RT-qPCR analysis. The viral load in the brain was also titrated by focus-forming assay. The dashed lines represent the limit of detection by focus-forming assay. Median viral titers: two-tailed Mann–Whitney test *P < 0.05; **P < 0.01; ns, not significant. **h.** Body weight change of hamsters inoculated intranasally with Sfull or Sdel virus. 6-8 week-old hamsters (n=6) were infected with Sfull or Sdel virus and the body weight was measured daily until day 14. Body weight: two-way ANOVA with Sidak's test; mean ± s.d.; *P < 0.05; **P < 0.01; ***, P < 0.001; ****P < 0.0001.

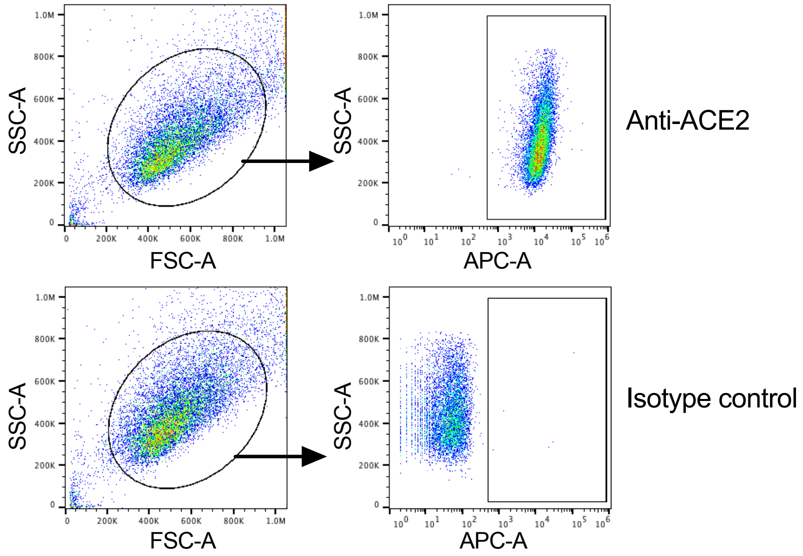

Supplementary Figure 8. Gating strategy for flow cytometry. Gating strategy for the flow cytometry presented in Figure 4b and c as follows: The A549-ACE2 or its gene-edited cells were gated with SSC-A vs FSC-A. The positive cells were gated with SSC-A vs APC-A. The cells stained with isotype control and secondary antibody were used as the control for gating.
