# Supplementary material for: A genome-wide CRISPR screen identifies host factors that regulate SARS-CoV-2 entry
Source: Nat Commun. 2021 Feb 11;12:961. doi: 10.1038/s41467-021-21213-4 (PMC7878750; doi:10.1038/s41467-021-21213-4)
Supplement: Supplementary file 3 — Description of Additional Supplementary Files [file 41467_2021_21213_MOESM3_ESM.pdf]

Description of Additional Supplementary Files

File Name: Supplementary Data 1
Description: List of genes and scores after MaGeck analysis (see Excel file). Data was obtained by deep-sequencing of sgRNAs from uninfected or survived cells.

File Name: Supplementary Data 2
Description: sgRNA sequences of genes selected for validation and other editing experiments (see Excel file).

File Name: Supplementary Data 3
Description: The complete list of primers used for plasmid contruction (see Excel file).
