# Supplementary material for: A genome-wide CRISPR screen identifies host factors that regulate SARS-CoV-2 entry
Source: Nat Commun. 2021 Feb 11;12:961. doi: 10.1038/s41467-021-21213-4 (PMC7878750; doi:10.1038/s41467-021-21213-4)
Supplement: Supplementary file 7 — Reporting Summary [file 41467_2021_21213_MOESM7_ESM.pdf]

nature research

Corresponding author(s): Rong Zhang

Last updated by author(s): Jan 7, 2021

Reporting Summary

Nature Research wishes to improve the reproducibility of the work that we publish. This form provides structure for consistency and transparency in reporting. For further information on Nature Research policies, see our Editorial Policies and the Editorial Policy Checklist.

Statistics

For all statistical analyses, confirm that the following items are present in the figure legend, table legend, main text, or Methods section.

n/a	Confirmed	
☐	☒	The exact sample size (*n*) for each experimental group/condition, given as a discrete number and unit of measurement
☐	☒	A statement on whether measurements were taken from distinct samples or whether the same sample was measured repeatedly
☐	☒	The statistical test(s) used AND whether they are one- or two-sided<br>*Only common tests should be described solely by name; describe more complex techniques in the Methods section.*
☒	☐	A description of all covariates tested
☒	☐	A description of any assumptions or corrections, such as tests of normality and adjustment for multiple comparisons
☐	☒	A full description of the statistical parameters including central tendency (e.g. means) or other basic estimates (e.g. regression coefficient) AND variation (e.g. standard deviation) or associated estimates of uncertainty (e.g. confidence intervals)
☐	☒	For null hypothesis testing, the test statistic (e.g. *F*, *t*, *r*) with confidence intervals, effect sizes, degrees of freedom and *P* value noted<br>*Give P values as exact values whenever suitable.*
☒	☐	For Bayesian analysis, information on the choice of priors and Markov chain Monte Carlo settings
☒	☐	For hierarchical and complex designs, identification of the appropriate level for tests and full reporting of outcomes
☒	☐	Estimates of effect sizes (e.g. Cohen's *d*, Pearson's *r*), indicating how they were calculated

Our web collection on statistics for biologists contains articles on many of the points above.

Software and code

Policy information about availability of computer code

Data collection	FASTX-Toolkit (http://hannonlab.cshl.edu/fastx_toolkit/); cutadapt 1.8.1; MAGeCK 0.5.0； Invitrogen Attune NxT, Operetta High Content Imaging System; FlexStation 3 (Molecular Devices); Odyssey CLx Imaging System
Data analysis	GraphPad Prism version 8.0.0; FlowJo v10.0.7; PerkinElmer Harmony high-content analysis software v4.9; ImageJ v2.0.0; Image Studio 4.0

For manuscripts utilizing custom algorithms or software that are central to the research but not yet described in published literature, software must be made available to editors and reviewers. We strongly encourage code deposition in a community repository (e.g. GitHub). See the Nature Research guidelines for submitting code & software for further information.

Data

Policy information about availability of data

All manuscripts must include a data availability statement. This statement should provide the following information, where applicable:

- Accession codes, unique identifiers, or web links for publicly available datasets
- A list of figures that have associated raw data
- A description of any restrictions on data availability

The authors declare that all relevant data supporting the findings of this study are available within the paper and its Supplementary information. The Supplemental Tables provide data for the CRISPR-Cas9 screen, statistical analysis. Source data are provided with this paper. The full-length genome sequence of SARS-CoV-2 nCoV-SH01 strain is deposited in GenBank (accession no. MT121215).

Field-specific reporting

Please select the one below that is the best fit for your research. If you are not sure, read the appropriate sections before making your selection.

- [x] Life sciences
- [ ] Behavioural & social sciences
- [ ] Ecological, evolutionary & environmental sciences

For a reference copy of the document with all sections, see nature.com/documents/nr-reporting-summary-flat.pdf

Life sciences study design

All studies must disclose on these points even when the disclosure is negative.

Sample size	The sample size of each experiment was indicated in the figure legends with n equals to 4 or above from two or more independent biological experiments. No sample size calculation was performed. Sample size is chosen based on the standard of the corresponding field.
Data exclusions	No data were excluded.
Replication	Data shown are an average of two or more independent experiments performed in duplicate or triplicate. Similar findings were obtained from all repeats.
Randomization	Animals used in the study were allocated randomly. Randomization is not relevant to other cell culture-based experiments. The same number of cells were used for the experiments. The experiments were well-controlled and there is no background difference between experimental groups.
Blinding	Since the experimental groups were conducted in parallel with the sample procedures, and data are quantitative, the investigators who conducted the cell culture and animal experiments were not blinded, but the data collection and analysis were performed carefully by two different investigators and were blinded for all the experiments.

Reporting for specific materials, systems and methods

We require information from authors about some types of materials, experimental systems and methods used in many studies. Here, indicate whether each material, system or method listed is relevant to your study. If you are not sure if a list item applies to your research, read the appropriate section before selecting a response.

Materials & experimental systems

n/a	Involved in the study	
[ ]	[x]	Antibodies
[ ]	[x]	Eukaryotic cell lines
[x]	[ ]	Palaeontology and archaeology
[ ]	[x]	Animals and other organisms
[x]	[ ]	Human research participants
[x]	[ ]	Clinical data
[x]	[ ]	Dual use research of concern

Methods

n/a	Involved in the study	
[x]	[ ]	ChIP-seq
[ ]	[x]	Flow cytometry
[x]	[ ]	MRI-based neuroimaging

Antibodies

Antibodies used	rabbit anti-COMMD3 (proteintech #26240-1-AP), rabbit anti-VPS35 (proteintech #10236-1-AP), rabbit anti-CCDC22 (proteintech #16636-1-AP), rabbit anti-NPC1 (proteintech #13926-1-AP), rabbit anti-NPC2 (proteintech #19888-1-AP), rabbit anti-CCDC53 (proteintech #24445-1-AP), rabbit anti-COMMD1 (proteintech #11938-1-AP), mouse anti-SNX27 (Abcam #ab77799), rabbit anti-SNX17 (proteintech, #10275-1-AP), rabbit anti-LDLR (proteintech, #10785-1-AP), rabbit anti-LRP1 (Abcam #ab92544), rabbit anti-SARS-Cov-2 spike S2 (Sino Biological #40590-T62), rabbit anti-β-actin (proteintech #20536-1-AP),Goat anti-mouse (sigma #A4416), goat anti-rabbit (thermo fisher #31460), goat anti-human (sigma #A6029),goat anti-rabbit IRDye 800CW secondary antibody (LI-COR #926-32211,), goat anti-rabbit IRDye 680RD secondary antibody (LI-COR #926-68071), goat anti-mouse IRDye 800CW secondary antibody (LI-COR #926-32210),Alexa Fluor 555 (Thermo #A-21424), anti-ACE2 antibody (Sino Biological #10108-RP01), goat anti-human IgG (H + L) conjugated with Alexa Fluor 647 (Thermo #A21445), anti-ACE2 antibody (Sino Biological #10108-RP01)
Validation	Commercial primary antibodies were validated by the manufacturers and validation statements are available on the manufacturer's website.<br>The validation of house-made mouse anti-SARS-CoV-2 nucleocapsid (N) protein serum for immunofluorescence staining was provided in the current study.

Eukaryotic cell lines

Policy information about cell lines

Cell line source(s)	Vero E6 (Cell Bank of the Chinese Academy of Sciences, Shanghai, China), HEK 293T (ATCC # CRL-3216), HeLa (ATCC #CCL-2), A549 (ATCC #CCL-185), and Calu-3 (Cell Bank of the Chinese Academy of Sciences, Shanghai, China).
Authentication	None of the cell lines used in the manuscript were authenticated by authors.
Mycoplasma contamination	All cell lines used were tested routinely and free of mycoplasma contamination.
Commonly misidentified lines (See ICLAC register)	None.

Animals and other organisms

Policy information about studies involving animals; ARRIVE guidelines recommended for reporting animal research

Laboratory animals	Six to ten week-old male golden Syrian hamsters were used.
Wild animals	No wild animals used.
Field-collected samples	No field-collected samples involved.
Ethics oversight	The animal experiment protocol has been approved by the Animal Ethics Committee of School of Basic Medical Sciences at Fudan University. The virus isolation from patient was approved by the Shanghai Municipal Health and Family Planning Commission.

Note that full information on the approval of the study protocol must also be provided in the manuscript.

Flow Cytometry

Plots

Confirm that:

- [x] The axis labels state the marker and fluorochrome used (e.g. CD4-FITC).
- [x] The axis scales are clearly visible. Include numbers along axes only for bottom left plot of group (a 'group' is an analysis of identical markers).
- [x] All plots are contour plots with outliers or pseudocolor plots.
- [x] A numerical value for number of cells or percentage (with statistics) is provided.

Methodology

Sample preparation	Common cell lines were collected from cell culture by using the TrypLE (Thermo #12605010). We didn't collect cells from tissues.
Instrument	Thermo, Attune™ NxT
Software	FlowJo vX.0.7
Cell population abundance	We did not sort our cells.
Gating strategy	Gating was used to eliminate debris and multiplet cells using forward and side scatter parameters (SSC-A/FSC-A). The viral S1 protein or ACE2 antibody bound cells were gated positive based on SSC-A/APC-A. The staining with isotype control was used as the control of gating. The gating strategy was demonstrated in Supplementary Figure 8.

- [x] Tick this box to confirm that a figure exemplifying the gating strategy is provided in the Supplementary Information.
